# Ultra-Proximal Jejunostomy Application after McKeown-Type Esophagectomy: A Retrospective Case-Series Study

**DOI:** 10.1155/2023/5874332

**Published:** 2023-08-02

**Authors:** Dongliang Lin, Zhendong Xu, Jinlong Huang, Wenshan Hong, Weiqing Zhang, Luoyu Lian

**Affiliations:** Department of Thoracic Surgery, Quanzhou First Hospital, Quanzhou, 362000 Fujian, China

## Abstract

**Background:**

Jejunostomy is the main form of enteral nutritional support after McKeown-type esophagectomy. However, this requires the jejunum to be secured to the abdominal wall, which can lead to catheter-related complications. Here, we present a new type of jejunostomy, ultra-proximal jejunostomy, which does not require fixation of the jejunum to the abdominal wall.

**Methods:**

Patients who underwent McKeown-type esophagectomy between January 2021 and March 2022 were included in this study. Postoperative outcomes of patients who underwent ultra-proximal jejunostomy are also presented.

**Results:**

Forty-three patients were able to receive enteral nutritional support via an ultra-proximal jejunostomy after McKeown-type esophagectomy, and no cases of enteral fistulas were observed. The pain in the left lower abdomen largely disappeared after the removal of the jejunostomy tube in all patients, and there was no difficulty in removing the tube. To date, none of these patients have experienced bowel obstruction or jejunal torsion.

**Conclusion:**

An ultra-proximal jejunostomy is a safe and feasible method and a better option for enteral nutrition support after McKeown-type esophagectomy.

## 1. Introduction

Surgery for esophageal cancer is traumatic, resulting in an inability to feed orally for a long time after surgery. Enteral nutritional support at the early postoperative stage has been accepted by most researchers [1]. Jejunostomy is the mainstream means of enteral nutritional support after esophageal cancer surgery and has several advantages over the nasojejunal tube [2]. However, many catheter-related complications (such as bowel obstruction and jejunal torsion) have occurred after jejunostomy, some of which required resolution with secondary surgery [3]. Catheter-related complications may be associated with jejunal fixation to the abdominal wall during fistulisation. Additionally, some scholars have proposed that the jejunal nutrient tube be led outside the abdominal wall via the round ligament of the liver during jejunostomy [4, 5]. However, this technique, which does not require jejunal fixation of the abdominal wall, is not widely used in China.

In this study, we present a new jejunostomy technique, that is, not fixed to the abdominal wall and is referred to as an ultra-proximal jejunostomy. Here, we describe the details of this technique and report the short-term outcomes of patients who underwent surgery at our hospital.

## 2. Materials and Methods

### 2.1. Patients

Patients who underwent McKeown-type esophagectomy at Quanzhou First Hospital between January 2021 and March 2022 were included in this study. Postoperative nutritional support was achieved using an ultra-proximal jejunostomy. All patients met the preoperative diagnostic criteria for esophageal cancer according to the Guidelines for Standardized Diagnosis and Treatment of Esophageal Cancer, and the diagnosis was confirmed by pathological examination. This retrospective case series was approved by the Ethics Committee of Quanzhou First Hospital (2020-202), and all participants provided written informed consent.

### 2.2. Operative Procedure

**Figure 1:**
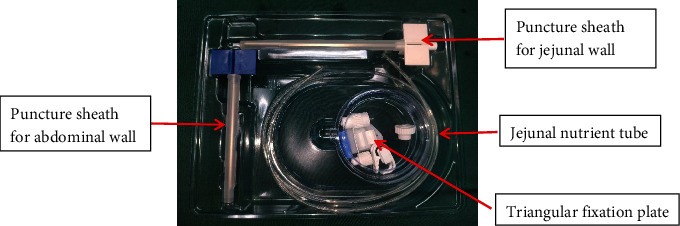
Jejunostomy device.

After completing the McKeown-type esophagectomy and anastomosis of the esophagus with the neck of the tubular stomach, an ultra-proximal jejunostomy was performed through the original epigastric incision. The jejunostomy device (Freka^®^FCJ Set FR9) was obtained from Huarui Pharmaceutical Co., Ltd. (Wuxi, China) and is shown in Figure 1.

**Figure 2:**
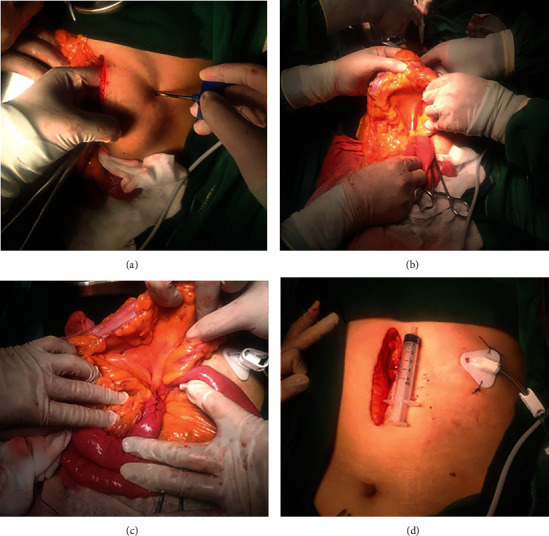
Ultra-proximal jejunostomy was performed after McKeown-type esophagectomy. (a) Jejunal nutrient tube passed through the skin into the abdominal cavity via a puncture device. (b) Jejunal nutrient tube passed into the root of transverse mesocolon through a puncture device. (c) Jejunal nutrient tube passed into the jejunum, which is 5 cm from Treitz's ligament. (d) Jejunal nutrient tubes were fixed to the skin of the left abdominal wall.

The puncture point of the abdominal wall was selected to be approximately 6 cm to the left of the incision and 2 cm below the costal margin to ensure that the corresponding peritoneum was free of intestinal adhesions. The puncture sheath was punctured through the skin into the peritoneal cavity, followed by the insertion of a jejunal nutrient tube along the puncture sheath through the skin into the peritoneal cavity (Figure 2(a)).

Next, the puncture sheath was punctured at the root of the transverse mesocolon near the ligament of Treitz, and the jejunal nutrient tube was passed through the transverse mesocolon along the puncture sheath (Figure 2(b)).

Subsequently, the puncture point was chosen to be 5 cm from the ligament of Treitz on the opposite mesenteric side of the jejunum. A puncture device was used to penetrate the intestinal wall under the serosa membrane to the distal jejunum, 3 cm before penetrating the intestinal cavity to place the jejunal nutrient tube.

Simultaneously, a 50 mL syringe was used to continuously fill the jejunal nutrient tube with water to smoothly advance the tube to the distal end, a length of approximately 40 cm. The puncture point of the intestinal wall was knotted with a single purse-string suture using a 1-0 silk thread. The seromuscular layer was sutured (with three stitches) along the intestinal wall on both sides of the jejunal nutrient tube, and the lateral wall of the jejunum was used to tunnel the jejunal nutrient tube 2 cm (Figure 2(c)).

Finally, the transverse colon was repositioned, the intra-abdominal jejunal tube was straightened from the outside of the abdominal wall, and the jejunal nutrient tube was fixed to the skin of the abdominal wall using a triangular fixation plate and clasp (Figure 2(d)).

### 2.3. Postoperative Management

Enteral nutrition via the jejunal nutrient tube was initiated on the first postoperative day using an infusion pump, and the gastrointestinal decompression tube was removed once the flatus passed through. On the first day, 500 mL of glucose sodium chloride was infused, followed by 500 mL of enteral nutritional emulsion (Ruinneg or Ruidai [patients with diabetes], Huarui Pharmaceutical Co., Ltd.) on the second day, and 1000 mL of enteral nutritional emulsion on the third day. The infusion rate and daily infusion volume were individually adjusted according to each patient's tolerance, generally reaching 1500-1700 mL/day (30 kCal/(kg day)). Warm water was administered first for patients without anastomotic leakage on the seventh day postoperatively. Liquid food was introduced on the eighth day, and semi-liquid food on the ninth day. The amount of enteral nutritional emulsion was reduced as appropriate based on the patient's food intake. Patients were discharged with the jejunal nutrient tube in place, which was removed during their outpatient review approximately three weeks later, depending on their eating status.

## 3. Results

### 3.1. Patients

Between January 2021 and March 2022, 43 patients (30 males and 13 females, aged 44-77 years, TNM stage I B to III C underwent McKeown-type esophagectomy at our hospital, after which postoperative nutritional support was achieved via ultra-proximal jejunostomy. The trial included six patients (13.95%) with carcinoma in the upper part of the esophagus, 21 patients (48.84%) in the middle, and 16 patients (37.21%) in the distal esophagus. Additionally, the trial included 38 patients (88.37%) with squamous carcinoma, 4 patients (9.30%) with adenocarcinoma, and 1 patient (2.33%) with adenosquamous carcinoma.

### 3.2. Postoperative Observations

The postoperative observations of all patients are as follows: (1) all patients completed the operation successfully without any fatalities; (2) all patients were able to receive enteral nutritional support via ultra-proximal jejunostomy after surgery; (3) no cases of postoperative intestinal fistula were observed; (4) all patients had their jejunal nutrient tubes removed normally after three weeks of oral feeding, and there were no cases of difficult extraction or post-extraction enterocutaneous fistula; (5) the pain in the left lower abdomen had essentially subsided after removing the jejunal nutrient tube in all patients (Numeric Rating Scale scores ranging from 0 to 1); and (6) as of April 27th, 2023, none of the patients experienced bowel obstruction or jejunal torsion from the jejunostomy.

## 4. Discussion

Esophageal cancer is a malignant tumor with a high incidence in China and often occurs in middle-aged and elderly individuals. Most patients are already in the middle and late stages of diagnosis, and surgery remains the primary means of treatment for esophageal cancer [6]. Although perioperative mortality rates for surgery were high in the past, they have now been considerably reduced owing to the development of nutritional support and antimicrobial drugs, along with improvements in surgical levels.

Postoperative nutritional support for patients with esophageal cancer can be achieved via intravenous or enteral routes. Enteral nutritional support has been accepted by most researchers because it contributes to restoring digestive tract function early, maintaining normal metabolism and mucosal barrier function in the intestine, preventing bacterial translocation, and promoting protein synthesis [7]. Currently, enteral nutrition support delivery options include nasojejunal and jejunostomy tube placement. Nasojejunal tube placement preserves the natural physiological anatomy of the intestinal tube and minimizes catheter-related complications. However, the disadvantages of this method include subjective pain experienced by patients and potential lung infections resulting from nausea, vomiting, asphyxia, and accidental aspiration. Additionally, patients often self-remove the nasojejunal tube, which may disrupt the delivery of enteral nutrition support. In contrast, jejunostomy tube placement has the advantages of greater comfort and improved patient compliance, thus enabling enteral nutrition to be implemented smoothly and facilitating postoperative nutritional recovery [8].

**Figure 3:**
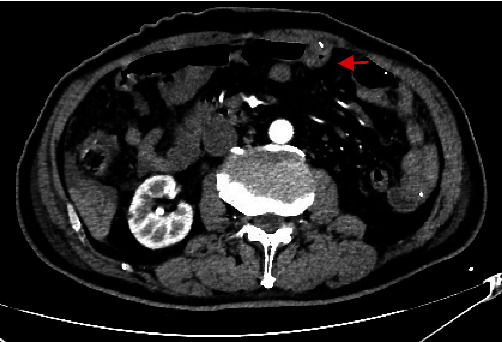
Conventional jejunostomy fixes the jejunum to the abdominal wall, altering the anatomical position of the normal intestinal tube.

In China, most researchers routinely recommend using jejunostomies to provide nutritional support after esophageal cancer surgery [9]. However, some scholars do not support jejunostomies [10, 11]. This is primarily due to the conventional method of jejunostomy placement, which requires suspension and fixation of the jejunum to the abdominal wall approximately 30 cm from the ligament of Treitz, leading to alterations in the normal anatomy and physiology of the intestinal canal (Figure 3). Additionally, this method carries the risk of developing an artificial internal hernia, bowel obstruction, and jejunal torsion, which may require mitigation through secondary surgery [12].

**Figure 4:**
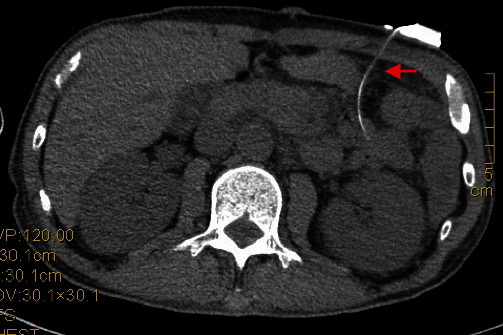
Ultra-proximal jejunostomy does not fix the jejunum to the abdominal wall, so that does not alter the anatomical position of the normal intestinal tube.

The main reasons for fixing the jejunum to the abdominal wall in conventional jejunostomy are as follows: (1) to avoid slipping of the jejunal nutrient tube from the lumen due to peristaltic movement of the intestine, causing enterocutaneous fistula and nutrient fluid to enter the abdominal cavity, and (2) to close the gap between the intestinal tube and peritoneum to reduce the occurrence of enterocutaneous fistula when the jejunal nutrient tube is removed. In this study, jejunostomy was improved as follows: (1) the jejunal puncture point was chosen 5 cm from the distal end of the ligament of Treitz. The jejunum was fixed at this point, and the jejunal nutrient tube was less likely to slip out of the intestinal canal because of the small change in position of the space produced by peristalsis; therefore, the jejunum did not need to be fixed to the abdominal wall. (2) The lateral wall of the jejunum was used to tunnel the nutrient tube for 2 cm, and the tube was passed through the root of the transverse mesocolon to the outside of the abdomen via the peritoneum. Because a sinus tract was formed, it was less likely to cause an enterocutaneous fistula in the long term (Figure 4). None of the patients developed an enterocutaneous fistula, and there were no complications related to tube removal, suggesting that this improved method was effective and safe.

Conventional jejunostomy has been associated with difficulty in withdrawing the jejunal nutrient tube, possibly because the tube must be secured with sutures. Ultra-proximal jejunostomy does not require sutures to secure the intestinal tube; therefore, there have been no cases of difficult extraction. It is also possible to shorten the duration of the jejunostomy procedure, which is another advantage of a proximal jejunostomy.

Patients who undergo conventional jejunostomy often experience long-lasting pain in the left lower abdomen, mainly in the vicinity of the abdominal fistula opening, particularly after eating. This may be related to the fixation of the jejunum to the abdominal wall. The pain in the left lower abdomen largely disappeared after removing the jejunostomy tube in patients with ultra-proximal jejunostomy, suggesting that this method, to some extent, could reduce postoperative left lower abdominal pain.

**Figure 5:**
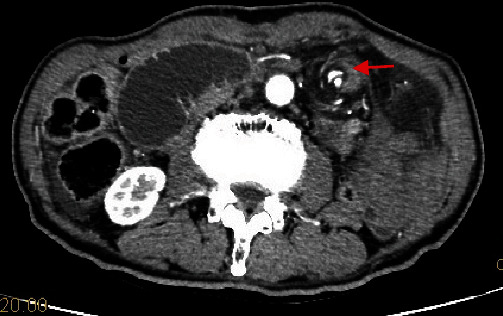
Abdominal computerized tomography shows jejunal torsion owing to the jejunum was fixed to the abdominal wall.

**Figure 6:**
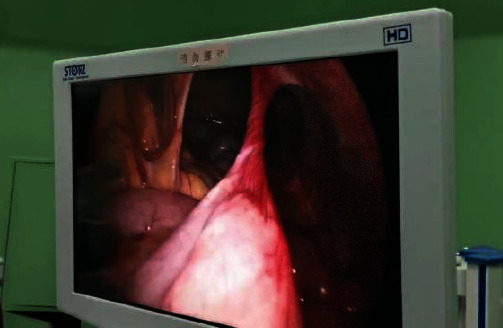
Intraoperative photos of jejunal torsion after esophageal cancer surgery.

A drawback of this study was that our study could not conclusively prove that ultra-proximal jejunostomy reduces the incidence of postoperative bowel obstruction and intestinal torsion due to its limited sample size and duration. However, most clinical cases of bowel obstruction and jejunal torsion are associated with the jejunum fixed to the abdominal wall (Figures 5 and 6). By contrast, the present method does not fix the jejunum to the abdominal wall intraoperatively, which reduces the incidence of postoperative bowel obstruction and jejunal torsion. This should be supported by additional case data.

In conclusion, an ultra-proximal jejunostomy is a secure and efficient approach for administering enteral nutrition to patients after McKeown-type esophagectomy. It not only reduces the duration of the procedure but also alleviates postoperative left lower abdominal pain. Furthermore, it is theoretically superior to conventional jejunostomy in terms of catheter-related complications, such as bowel obstruction and jejunal torsion. Therefore, it is recommended for clinical use.

## Data Availability

Data supporting this research article are available from the corresponding author or the first author on reasonable request.
